# Distribution in microbial genomes of genes similar to *lodA* and *goxA* which encode a novel family of quinoproteins with amino acid oxidase activity

**DOI:** 10.1186/s12864-015-1455-y

**Published:** 2015-03-24

**Authors:** Jonatan C Campillo-Brocal, María Dolores Chacón-Verdú, Patricia Lucas-Elío, Antonio Sánchez-Amat

**Affiliations:** Department of Genetics and Microbiology, University of Murcia, Campus de Espinardo, Murcia 30100 Spain

**Keywords:** L-amino acid oxidase, Quinone cofactor, Post-translational modification, Lysine oxidase, Glycine oxidase

## Abstract

**Background:**

L-Amino acid oxidases (LAOs) have been generally described as flavoproteins that oxidize amino acids releasing the corresponding ketoacid, ammonium and hydrogen peroxide. The generation of hydrogen peroxide gives to these enzymes antimicrobial characteristics. They are involved in processes such as biofilm development and microbial competition. LAOs are of great biotechnological interest in different applications such as the design of biosensors, biotransformations and biomedicine.

The marine bacterium *Marinomonas mediterranea* synthesizes LodA, the first known LAO that contains a quinone cofactor. LodA is encoded in an operon that contains a second gene coding for LodB, a protein required for the post-translational modification generating the cofactor. Recently, GoxA, a quinoprotein with sequence similarity to LodA but with a different enzymatic activity (glycine oxidase instead of lysine-ε-oxidase) has been described. The aim of this work has been to study the distribution of genes similar to *lodA* and/or *goxA* in sequenced microbial genomes and to get insight into the evolution of this novel family of proteins through phylogenetic analysis.

**Results:**

Genes encoding LodA-like proteins have been detected in several bacterial classes. However, they are absent in *Archaea* and detected only in a small group of fungi of the class *Agaromycetes*. The vast majority of the genes detected are in a genome region with a nearby *lodB*-like gene suggesting a specific interaction between both partner proteins.

Sequence alignment of the LodA-like proteins allowed the detection of several conserved residues. All of them showed a Cys and a Trp that aligned with the residues that are forming part of the cysteine tryptophilquinone (CTQ) cofactor in LodA. Phylogenetic analysis revealed that LodA-like proteins can be clustered in different groups. Interestingly, LodA and GoxA are in different groups, indicating that those groups are related to the enzymatic activity of the proteins detected.

**Conclusions:**

Genome mining has revealed for the first time the broad distribution of LodA-like proteins containing a CTQ cofactor in many different microbial groups. This study provides a platform to explore the potentially novel enzymatic activities of the proteins detected, the mechanisms of post-translational modifications involved in their synthesis, as well as their biological relevance.

**Electronic supplementary material:**

The online version of this article (doi:10.1186/s12864-015-1455-y) contains supplementary material, which is available to authorized users.

## Background

L-Amino acid oxidases (LAOs) are enzymes which oxidize amino acids releasing the corresponding ketoacid, ammonium and hydrogen peroxide. They have been found in different microbial groups including bacteria [1,2] although the most studied members of this group are the LAOs present in snake venoms [3]. Enzymes oxidizing amino acids are of great biotechnological interest in many different fields such as the design of biosensors, processes of biotransformation and biomedicine [1]. However, in some cases their use is limited by the difficulties of their recombinant expression [4]. In addition to their biotechnological value, the unraveling of novel metabolic pathways of amino acids is of great interest since, apart from their essential roles in primary metabolism, these pathways are also related to the secondary metabolism in processes such as the synthesis of pigments, antibiotics, etc.

The marine gammaproteobacterium *Marinomonas mediterranea* synthesizes LodA and GoxA, the first two described LAOs that do not contain FAD but a quinone cofactor. LodA was the first enzyme described with L-lysine-ε-oxidase activity [5]. It received a new number by the Enzyme Commission (EC 1.4.3.20). It has been demonstrated recently that the cofactor of LodA is cysteine tryptophylquinone (CTQ) [6]. CTQ cofactor was described for the first time in a quinohemoprotein amine dehydrogenase (QHNDH) [7]. Protein quinone cofactors are generated by post-translational modification of amino acid residues in the protein [8]. In the case of LodA, the modification involves the generation of a quinone from tryptophan 581 and its linking to cysteine 516. In this regard, it has been demonstrated that LodB, a flavoprotein encoded in the same operon as LodA, is involved in the post-translational modification that generates the quinone cofactor [9,10].

LodA and similar proteins play a role in microbial biofilm development and cell dispersal from the biofilm. This dispersion takes place after cell death of part of the population which is mediated by the hydrogen peroxide released [11]. In the *Pseudoalteromonas tunicata* autolytic protein AlpP, lysine oxidase activity was observed. However, in other cases such as *Chromobacterium violaceum* and *Caulobacter crescentus*, although hydrogen peroxide was released, the substrate of the activity was not reported [11].

Genome sequencing of *M. mediterranea* revealed that it contains two other genes with similarity to *lodA*. In both cases they are followed by a gene with similarity to *lodB* [12]. One of those *lodA*-like genes codes for GoxA, a quinoprotein with glycine oxidase activity and properties, such as high substrate specificity, clearly different to other flavoproteins with glycine oxidase activity [13]. Those observations suggest that proteins similar to LodA could constitute a reservoir of novel enzymatic activities with potential biotechnological interest. A possibility is that they could oxidize different amino acids, or some structurally related compounds.

The aim of this study was to study the distribution of genes encoding proteins similar to LodA and/or GoxA in sequenced microbial genomes and to get insight into the evolution of this novel family of proteins through phylogenetic analysis. We show that proteins similar to LodA are present in several classes of *Bacteri*a, absent in *Archaea* and detected only in a small group of fungi of the class *Agaromycetes*. Those proteins can be clustered in different groups, with LodA and GoxA in distinct groups, indicating that the groups observed may inform the enzymatic activity of the protein clusters.

## Results and discussion

### Identification of genes similar to *lodA* and/or *goxA* in microbial genomes

Using as query the sequence of the two *M. mediterranea* quinoproteins with amino acid oxidase activity: LodA (accession number AAY33849) [5] and GoxA (accession number ADZ90918) [13], BLASTP search was performed against sequenced microbial genomes deposited in the Integrated Microbial Genomes database as of January 8, 2014. Using GoxA as query and a cut-off limit for the E-value of 1e-10, 170 genes encoding proteins with similarity to it were detected. With LodA a slightly smaller number of genes were observed. Since all of them were included in the former group, those 170 genes were used in further analysis. Two genes were not included in the final analysis because they encode hypothetical proteins which seemed to be truncated. This gave a final selection of 168 genes named in this study as *lodA*-like genes or belonging to the *lodA* family (Additional file 1: Table S1).

Regarding the distribution of genes encoding proteins of the LodA family in microbial genomes it is important to indicate that the number of microbial genomes sequenced show an uneven distribution, with some microbial groups more represented than others (Table 1). For that reason, the number of genomes with genes of the *lodA* family is generally expressed in this paper as percentage of the total of microbial genomes sequenced in the group considered. Genes encoding proteins of the LodA family were not detected in *Archaea* (602 genomes sequenced at the time of the analysis). In *Eukarya* they have been detected in just 4 strains, with *Gymnopus luxurians* containing two copies (Table 2). Although the number of strains is low, in terms of percentage, they represent the 1.9% of the total number of *Eukarya* sequenced and 26.6% of the *Basydiomycota* (Table 1).

**Table 1:** **Distribution of** ***lodA*** **-like genes in microbial genome sequences deposited with IMG database as of January 2014**

**Taxon**	**Genomes with** ***lodA*** **-like genes**	**Percentage**
01 ***Archaea*** **(602)**	0	<0.16
01 ***Bacteria*** **(14983)** ^**1**^	140	0.94
02 *Acidobacteria* (34)	1	2.94
02 *Actinobacteria* (1401)	13	0.93
02 *Bacteroidetes* (598)	11	1.83
02 *Chloroflexi* (90)	2	2.22
02 *Cyanobacteria* (313)	9	2.88
02 *Firmicutes* (3658)	1	0.03
03 *Bacilli* (2695)	1	0.04
04 *Bacillales* (1103)	1	0.09
04 *Lactobacillales* (1592)	0	<0.06
03 *Clostridia* (799)	0	<0.13
03 *Erysipelotrichia* (33)	0	<3.03
03 *Negativicutes* (86)	0	<1.16
03 unclassified (45)	0	<2.22
02 *Planctomycetes* (40)	3	7.50
02 *Proteobacteria* (7187)	100	1.39
03 *Alphaproteobacteria* (1233)	37	3.00
03 *Betaproteobacteria* (774)	19	2.45
03 *Deltaproteobacteria* (183)	3	1.64
03 *Epsilonproteobacteria* (469)	0	<0.21
03 *Gammaproteobacteria* (4466)	41	0.92
03 *Zetaproteobacteria* (19)	0	<5.26
03 unclassified (43)	0	<2.33
01 ***Eukarya*** **(203)** ^**2**^	4	1.97
02 *Basidiomycota* (15)	4	26.66

The numbers in brackets indicate the number of genomes sequenced in each taxon. The number before each taxon is 01 for Domain, 02 for Phylum, 03 for Class and 04 for Order.

^1^Phyla among *Bacteria* without *lodA*-like genes: *Aquificae* (22), *Armatimonadetes* (9), *Atribacteria* (1), *Caldiserica* (2), *Candidatus Saccharibacteria* (5), *Chlamydiae* (100), *Chlorobi* (25), *Chrysiogenetes* (2), *Deferribacteres* (7), *Deinococcus-Thermus* (43), *Dictyoglomi* (2), *Elusimicrobia* (3), *Fibrobacteres* (9), *Fusobacteria* (47), *Gemmatimonadetes* (8), *Lentisphaerae* (3), *Nitrospinae* (1), *Nitrospirae* (19), *Poribacteria* (11), *Spirochaetes* (414), *Synergistetes* (20), *Tenericutes* (146), *Thermodesulfobacteria* (6), *Thermotogae* (40), *Verrucomicrobia* (34), Candidate division CD12 (1), Candidate division EM 3 (2), unclassified (680).

^2^Phyla among *Eukarya* without *lodA*-like genes: *Apicomplexa* (12), *Ascomycota* (77), *Bacillariophyta* (2), *Blastocladiomycota* (1), *Chlorophyta* (8) *Chytridiomycota* (2), *Microsporidia* (5), *Neocallimastigomycota* (4), unclassified (26).

**Table 2:** **Microbial genomes deposited with IMG as of January 2014 with more than one copy of genes encoding proteins similar to LodA/GoxA**

**Genome Name**	**Phylum**	**Class**	**Phylogenetic Group of LodA-like proteins**		
*Tenacibaculum ovolyticum* DSM 18103	*Bacteroidetes*	*Flavobacteria*	II	IIIA	
*Kordia algicida* OT-1	*Bacteroidetes*	*Flavobacteria*	II	IVB	
*Thalassobaculum salexigens* DSM 19539	*Proteobacteria*	*Alphaproteobacteria*	IIB	IIB	
*Bradyrhizobium japonicum* USDA 38 and USDA 6	*Proteobacteria*	*Alphaproteobacteria*	ID	IVA	
*Nitrobacter hamburgensis* X14	*Proteobacteria*	*Alphaproteobacteria*	ID	None	
*Xanthobacter* sp. 126	*Proteobacteria*	*Alphaproteobacteria*	IB	None	
*Citreicella* sp. SE45	*Proteobacteria*	*Alphaproteobacteria*	IB	IIIB	
*Burkholderia* sp. BT03	*Proteobacteria*	*Betaproteobacteria*	III	None	
*Chitinimonas koreensis* DSM 17726	*Proteobacteria*	*Betaproteobacteria*	ID	III	
*Cellvibrio japonicus* Ueda107	*Proteobacteria*	*Gammaproteobacteria*	IB	III	
*Marinomonas mediterranea* MMB-1	*Proteobacteria*	*Gammaproteobacteria*	IA	IIB	III
*Oceanospirillum beijerinckii* DSM 7166	*Proteobacteria*	*Gammaproteobacteria*	IB	III	
*Pseudoalteromonas citrea* NCIMB 1889	*Proteobacteria*	*Gammaproteobacteria*	IA	II	IIIA
*Pseudoalteromonas luteoviolacea* 2ta16	*Proteobacteria*	*Gammaproteobacteria*	IA	IB	
*Pseudoalteromonas rubra* ATCC 29570	*Proteobacteria*	*Gammaproteobacteria*	IB	IIIA	
*Pseudoalteromonas flavipulchra* 2ta6, JG1	*Proteobacteria*	*Gammaproteobacteria*	IA	IIIA	
*Pseudoalteromonas piscicida* ATCC 15057 and JCM 20779	*Proteobacteria*	*Gammaproteobacteria*	IA	IIIA	
*Rheinheimera* sp. A13L	*Proteobacteria*	*Gammaproteobacteria*	IA	IIIA	
*Gymnopus luxurians* FD-317 M1	*Basidiomycota*	*Agaricomycetes*	V	V	

Most of the genes encoding proteins of the LodA family were found in *Bacteria* (Table 1). In this domain they were detected in, approximately, the 0.94% of the genomes sequenced. In most of the bacterial groups the percentage of bacteria with those genes was around 1-3%, with some exceptions. In the case of *Firmicutes*, out of 3658 genomes there was a single genome identified (*Paenibacillu*s *pinihumi*) containing a *lodA* gene. Two groups of microorganisms with a high number of genomes sequenced but with no *lodA*-like genes were *Spirochaeta* and *Tenericutes* (414 and 146 genomes respectively). Most of the *lodA* family genes were detected in *Proteobacteria* (121 genes out of 168). However, this seems to be the result of the high number of genomes sequenced in this group, since the percentage of *Proteobacteria* with *lodA* genes is on the average (1.39%). Among *Proteobacteria*, they were most abundant in *Alpha* and *Betaproteobacteria* (3 and 2.45% respectively). They were not found in *Epsilonproteobacteria* (0 out of 469 genomes). The percentage in *Gammaproteobacteria* was 0.94%.

According to their phylogenetic distribution, *lodA*-like genes seem to have an ancient origin since they are detected in a wide range of bacteria. In contrast, they are detected in a small number of fungi which are phylogenetically related since all of them belong to the class *Agaromycetes*. A possible explanation for this distribution could be that the acquisition of the gene in fungi took place through a process of horizontal gene transfer from a bacterium. The phylogenetic analysis performed in this study (see below) does not make it possible to suggest the bacterial origin of the fungal genes since the most similar proteins belong to different bacterial groups.

The 168 genes selected were distributed in 144 different microbial genomes since several microorganisms contained more than one copy (Table 2). Only two marine gammaproteobacteria, *Marinomonas mediterranea* MMB-1, order *Oceanospirillales*, and *Pseudoalteromonas citrea,* order *Alteromonadales,* showed three copies of those genes. In fact, genes of the *lodA* family are common in both genera. 4 out of the 5 *Marinomonas* genomes sequenced contained this kind of genes, although only *M. mediterranea* contained more than one copy. In the genus *Pseudoalteromonas*, about 50% of the strains sequenced showed genes of the *lodA* family. Moreover, 6 out of 8 of those genomes showed two or more copies.

Interestingly, no known human or animal pathogen contains genes of the *lodA* family. On the contrary, many of the microorganisms with that kind of genes have been isolated from the microbiota of plants or interact with them. For example, this is the case of many symbiotic *Alphaproteobacteria* such as *Bradyrhizobium.* In *Gammaproteobacteria* there are also many examples of microorganisms associated with algae or plants such as *M. mediterranea*, which is a member of the microbiota of the seagrass *Posidonia oceanica* [14]*.* These observations suggest the possibility of an ecological role of this kind of enzymes in the interaction between the plant and its associated microbiota, or in the growth of the microorganisms on the surface of the plant. In this regard, it has been observed that in several microorganisms these LodA-like proteins are involved in biofilm differentiation and dispersal [11].

### Detection of *lodB*-like genes in genomes with genes of the *lodA* family

**Figure 1:**
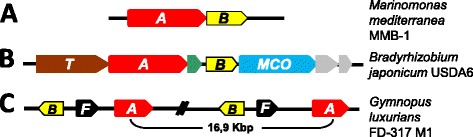
**Genome organization around the** ***lodA*** **-like genes (** ***A*** **in red) and** ***lodB*** **-like genes (** ***B*** **in yellow) in selected genomes. A)** *lod* operon in *M. mediterranea* [15]*.* **B)** *Bradyrhizobium japonicum* USDA6 operon containing *lodA* and *lodB*-like genes separated by a small gene shown in green. The gene in brown (marked with a *T*) encodes a protein containing the domain pfam00264 (tyrosinases). The product of the blue gene contains the domains pfam07731 and pfam07732 characteristic of multicopper oxidases (*MCO*). **C)** *Gymnopus luxurians* FD-317 M1 genome organization showing the two copies of *lodA* and *lodB*-like genes. The genes in black (marked with an *F*) encode proteins with the bacterial Ferritin-like domain (PF12902).

*lod* and *gox* operons contain, immediately downstream to the gene coding for the protein of the LodA family, a second gene coding for a putative flavoprotein (Figure 1A). In the *lod* operon this protein was named LodB and it has been demonstrated that it is required for the post-translational modification generating active LodA [9,10]. In all but one of the genomes analyzed, similarly to *lod* and *gox* operons, it has been possible to detect downstream the *lodA*-like gene, or very close in the genome, a *lodB*-like gene showing the conserved COG0644 described in flavoproteins (Additional file 2: Table S2).

The only genome in which no *lodB*-like gene could be detected close to the *lodA*-gene was *Cryptosporangium arvum.* In two other cases, the lack of the *lodB*-like gene could be accidental. In relation to the *lodA*-like gene CSE45_2361 from *Citreicella* sp. SE45, there is a large intergenic region downstream that gene. The examination of this sequence allowed the detection in direction 3’-5’ of a non-annotated ORF which codes for a protein bearing similarity to LodB-like proteins. In *Nitrolacentus hollandicus* LD the genome sequencing was incomplete and the *lodA*-like gene is at the end of a contig, so it was not possible to locate the *lodB*-like gene. However, a gene in another contig with accession number 2520384730 is a good candidate.

There are some modifications to the general pattern of a *lodB*-like gene located downstream the *lodA*-like gene. For instance, in 10 genomes (four of them *Bradyrhizobium*) *lodB*-like genes are located downstream to the *lodA*-like gene but separated from it by an additional small gene with a size between 138–142 amino acids (Additional file 2: Table S2 and Figure 1B). These genes code for small hypothetical proteins showing higher than 50% amino acid identity between them. These proteins do not possess any conserved domain. Since, as far as we know, no protein of this group has been characterized, their function remains unknown. Interestingly, the genomic organization suggests that all of the operons containing those small genes also include other genes coding for hypothetical tyrosinases and multicopper oxidases (Figure 1B). *M. mediterranea* also synthesizes tyrosinase and multicopper oxidase enzymes which are co-regulated with LodA, although they are not encoded by genes located in the same operon [16]. These observations suggest some possible functional relationship between all those enzymes.

With regard to exceptions to the general organization discussed above, in *Acidovorax avenae* RS-1 and in one *lodA*-like gene of *Oceanospirillum beijerinckii* DSM7166 (H579DRAFT_00201), the *lodA*-like genes are followed by two genes with similarity to flavoproteins. The comparison of these proteins with LodB revealed that the first one shows high similarity to the N-terminal region of LodB, while the second one is similar to the C-terminal region. For example, the product of *Oceanospirillum beijerinckii* DSM7166 gene H579DRAFT_00202 showed 47% similarity to residues 4–118 of LodB and the product of H579DRAFT_00203 showed 42% similarity to residues 130–160 of LodB. These observations suggest as the most likely explanation that those *lodB*-like genes are the result of the division of a previous gene.

In the five fungal *lodA*-like genes, the *lodB-*like genes were located in the opposite orientation. It is also worth mentioning that in these cases a gene coding for a ferritin-like protein was also detected close in the genome or between them (Figure 1C). Apart from fungi, in this study it has been also detected that *lodA* and *lodB*-like genes are in opposite orientations in the genome of the actinobacterium *Actinoplanes globisporus*.

The observation of the conservation of *lodB*-like genes associated to *lodA*-like genes in all but one of the genomes analyzed, suggests a strong selective force to maintain that association. Results from our group showed that each LodB-like protein might specifically be involved in the post-translational modification of its partner protein [17].

### Sequence analysis of proteins of the LodA family

**Figure 2:**
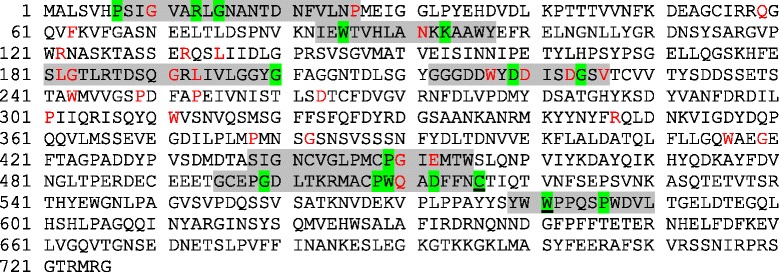
**Sequence analysis of LodA-like proteins.** Marked on the sequence of LodA the residues conserved in 100% of the proteins are shown highlighted in green. Of those, the two underlined are the residues that are modified to generate the cofactor. Other residues conserved in more than 90% of the selected proteins are shown in red. Several domains proposed to be conserved in LodA-like proteins are highlighted in grey.

In terms of sequence similarities, the alignment of the proteins similar to LodA detected in this study revealed several conserved residues in all of them (Figure 2). Interestingly, LodA C516 and W581, which have been described as the residues post-translationally modified to generate the quinone cofactor cysteine tryptophylquinone [6], aligned with Cys and Trp in all of the LodA-like proteins, suggesting that they possess the same quinone cofactor. In fact, modeling of GoxA supports that this protein contains CTQ [17]. The other residues conserved in these proteins could be involved in common processes to all of those proteins, being a possibility that they are involved in the generation of the quinone cofactor or in the catalytic activity of the enzymes.

Most of the proteins of the LodA family showed a size between 600 and 700 amino acids (Additional file 1: Table S1). In fact the average size is 738 amino acids, which is very close to the 726 amino acids of LodA. However, there are proteins with only 481 amino acids, such as A3CEDRAFT_0690 from *Amycolatopsis balhimycina* DSM 44591, and others with much larger sizes such as YY3DRAFT_04971 from *Rhizobium* sp. STM6155 with 1413 amino acids.

The proteins with larger sizes seem to be the result of gene fusions since, in addition to the sequence characteristics of the LodA family, they show conserved domains described in other proteins (Table 3) [18]. Some of these proteins’ N-terminal regions have similarity to the conserved domain pfam00199 characteristic of catalases. Those sequences also match the Conserved Domain CD08152 described in protein families related to the uncharacterized y4iL protein of *Rhizobium* sp. NGR234 which share the catalase fold and bind to heme, although they do not necessarily have catalase activity [19]. Five other proteins show in their N-terminal region sequences with similarity to the conserved domain pfam14518, described in iron-containing redox enzymes. Finally, two proteins from the actinobacteria *Amycolatopsis vancoresmycina* and *Cryptosporangium arvum* show the von Willebrand factor type A (vWA) domain (pfam13519). This domain was first described in the blood coagulation protein von Willebrand factor (vWF) but it has been described in other proteins. It is involved in different cellular processes that imply surface interactions mediated by a metal ion dependent adhesion site termed as the MIDAS motif [20]. The determination of the relevance of those additional conserved domains in proteins of the LodA family will require experimental work aimed at characterizing those proteins.

**Table 3:** **Products of the** ***lodA*** **-like genes showing conserved domains in addition to the characteristic sequences of the LodA family of proteins**

**Genome**	**gene_oid**	**Locus Tag**	**AA Seq Length**	**Pfams**
*Actinoplanes globisporus* DSM 43857	2515244410	A3CQDRAFT_07977	985	pfam14518
*Burkholderia* sp. BT03	2536908549	PMI06_03990	1409	pfam14518
*Calothrix* sp. PCC 7103	2507474092	Cal7103DRAFT_00009910	1049	pfam14518
*Paenibacillus pinihumi* DSM 23905	2524187775	H583DRAFT_01923	1099	pfam14518
*Rhizobium* sp. STM6155	2513599306	YY3DRAFT_04971	1413	pfam14518
*Acinetobacter gyllenbergii* MTCC 11365	2546621803	L293_0743	1008	pfam00199
*Acinetobacter* sp. NBRC 100985	2533901541		1008	pfam00199
*Acinetobacter tjernbergiae* DSM 14971	2518262899	C502DRAFT_01575	1006	pfam00199
*Azospirillum lipoferum* 4B	2512035869	AZOLI_p50417	999	pfam00199
*Azospirillum* sp. B510	646556648	AZL_e04100	1004	pfam00199
*Burkholderia* sp. BT03	2563064361	PMI06_008734	963	pfam00199
*Cupriavidus* sp. UYPR2.512	2514031881	A3A5DRAFT_06866	1025	pfam00199
*Flavobacterium soli* DSM 19725	2523123554	G508DRAFT_03147	1072	pfam00199
*Massilia timonae* CCUG 45783	2532942463	HMPREF9710_03282	979	pfam00199
*Microcystis aeruginosa* PCC 9701	2535024168		990	pfam00199
*Oceanospirillum beijerinckii* DSM 7166	2524095414	H579DRAFT_00201	973	pfam00199
*Pseudoalteromonas rubra* ATCC 29570	2541428757	PRUB_24676	1039	pfam00199
*Pseudoalteromonas* sp. BSi20495	2540458794		1000	pfam00199
*Pseudoalteromonas* sp. Bsw20308	2540452162	D172_1358	1000	pfam00199
*Ralstonia solanacearum* MolK2	2541798314		1000	pfam00199
*Ralstonia solanacearum* Po82	651230827	RSPO_m00447	999	pfam00199
*Rhizobium leguminosarum* bv. *viciae* 128C53	2515651856	B062DRAFT_04548	1004	pfam00199
*Sphingomonas* sp. S17	651582060	SUS17_588	986	pfam00199
*Streptomyces afghaniensis* 772	2546772914	STAFG_1983	999	pfam00199
*Streptomyces purpureus* KA281, ATCC 21405	2516519010	StrpuDRAFT_3616	993	pfam00199
*Tenacibaculum ovolyticum* DSM 18103	2523672835	H518DRAFT_02976	1061	pfam00199
*Amycolatopsis vancoresmycina* DSM 44592	2546378692	H480_25957	1114	pfam13519
*Cryptosporangium arvum* YU 629-21	2510402938	CryarDRAFT_3973	1026	pfam13519

### Phylogenetic analysis of LodA-like proteins

**Figure 3:**
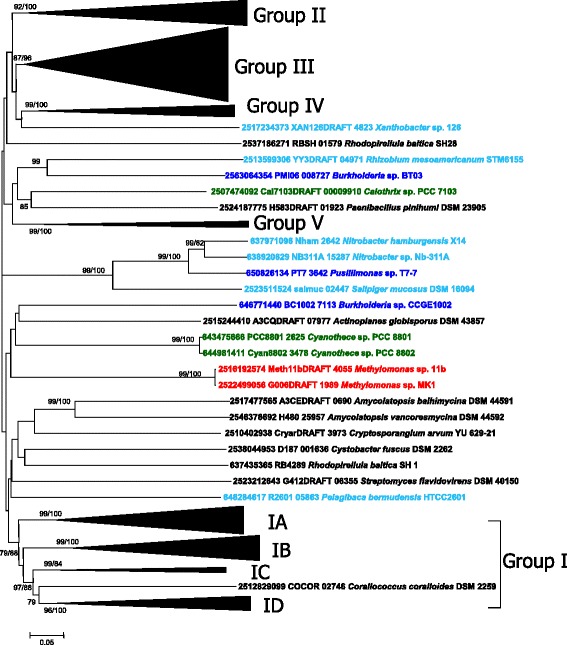
**Phylogenetic relationships of LodA-like proteins.** The tree was constructed using the Neighbor-Joining method built in the MEGA6 program. The distances between the proteins were computed using the p-distance method and are in the units of the number of amino acid differences per site. Numbers at branches indicate bootstrap values higher than 70% for both Neighbor-Joining and Maximum Likelihood trees. The nonclustered *Gammaproteobacteria* are indicated in red, *Alphaproteobacteria* in light blue, *Betaproteobacteria* in dark blue and photosynthetic microorganisms in green.

Once aligned, LodA-like proteins were subjected to phylogenetic analysis using Neighbor-Joining (NJ) and Maximum Likelihood (ML) methods available in the MEGA program [21]. In this study we have clustered the LodA-like proteins in several groups that met the criterion of being supported by bootstrap values higher than 70% in both phylogenetic analyses. As shown in Figure 3, five groups that included most of the proteins could be established. With the criterion used, other proteins could not be clustered (Figure 3). Among those are included the proteins synthesized by the actinobacteria *Amylocatopsis vancoresmycina* and *Cryptosporangium arvum* that show the conserved domain pfam13519 (vWA domain) previously mentioned. In the next paragraphs the characteristics of the different groups will be discussed.

#### Group I

**Figure 4:**
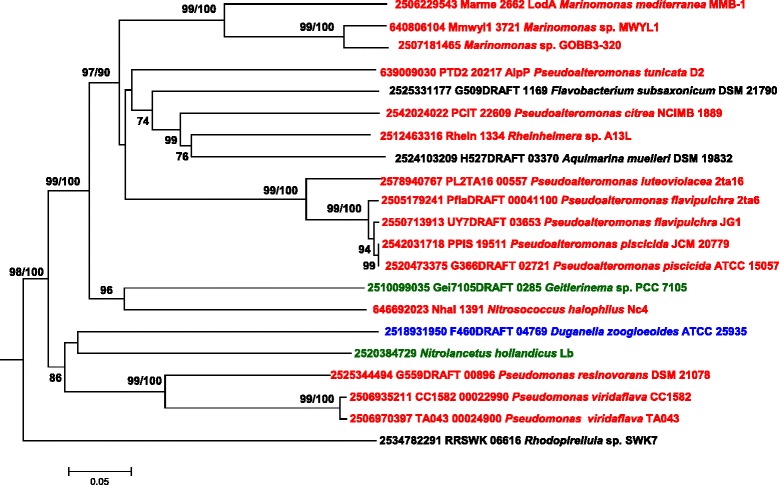
**Phylogenetic relationships of LodA-like proteins in the group IA.** The tree was constructed using the Neighbor-Joining method built in the MEGA6 program. The evolutionary distances were computed using the p-distance method and are in the units of the number of amino acid differences per site. Numbers at branches indicate bootstrap values > 70% for both Neighbor-Joining and Maximum Likelihood trees. Color codes for taxonomic groups as in Figure 3.

Group I contains a total of 56 proteins and could be divided in several subgroups as shown in Figure 3. Group IA contains *M. mediterranea* LodA, the first described enzyme with lysine-ε-oxidase activity [5], as well as *P. tunicata* AlpP in which lysine-ε-oxidase activity has been also demonstrated [11] (Figure 4). Both LodA and AlpP were initially described as antimicrobial proteins [22,23]. Interestingly, group IA includes proteins from many microorganisms, many of them in the genus *Pseudoalteromonas,* for which the synthesis of antimicrobial proteins has been reported. In *P. flavipulchra* JG1 two genes of the *lodA* family are detected, one of them (UY7DRAFT 03653) belongs to the group IA. It was demonstrated that the product of this gene is the protein PfaP which is an antimicrobial protein with high similarity to AlpP and LodA [24]. In another strain of the same species an antimicrobial protein was detected with sequence similarity to PfaP. It showed L-amino acid oxidase activity, but in this case it was able to oxidize not only L-lysine but also other amino acids such as L-Met, Gln, Leu, etc. [25]. An L-amino acid oxidase with similar broad substrate range (Met, Gln, Leu, Phe, Glu, Trp, etc.) has been described in several *P. luteoviolacea* strains whose genome has not been sequenced yet [26]. At the time of preparation of this manuscript there were two *P. luteoviolacea* strains whose genome had been sequenced. Strain B did not show any gene of the *lodA* family, although it is important to bear in mind that it has been proposed that this strain was misclassified and should be placed in a different species [27]. In strain 2ta16, two genes were detected, one in the group IA and the second in the group IB. As far as we know, no antimicrobial activity has been reported in this strain. Accordingly, for this species it is not possible to establish a relationship between the antimicrobial proteins and the *lodA*-like genes detected. With regards to other genera, in *Rheinheimera aquatica* strain GR5 lysine oxidase activity was demonstrated for a protein with a peptide fragment with high similarity to LodA and AlpP [28,29]. That fragment is also similar to the product of the gene Rhein1334 belonging to group IA from the sequenced *Rheinheimera* sp. strain A13L (Figure 4). The existence of this gene has been previously reported indicating that it could encode a lysine-ε-oxidase [30]. Interestingly this strain contains a second *lodA*-like gene that is phylogenetically distant (group III). Overall, our results indicate that proteins in the group IA show L-amino acid oxidase activity and that this capacity confers to them antimicrobial properties.

Apart from group IA, with the exception of the gene of the deltaproteobacterium *Corallococcus coralloides*, the other genes in group I could be associated to different clusters (IB, IC and ID) (Figure 3, Additional file 3: Figure S1 and Additional file 4: Figure S2). Group ID (Additional file 4: Figure S2) is particularly interesting since it contains the operons previously mentioned in which the *lodA*-like gene is separated from the *lodB*-like gene by a small gene (Figure 1B). *Saccharophagus degradans* 2–40 is the only microorganism in this group in which there is no small gene between *lodA*-like and *lodB*-like. Operons encoding proteins of group ID also contained genes encoding hypothetical tyrosinases and multicopper oxidases. The possible relationship between these two copper oxidases with LodA-like proteins is noteworthy since *M. mediterranea* also synthesizes a tyrosinase and a multicopper oxidase, distantly located in the genome, which are co-regulated with LodA [16]. In any case, the similarity between the operons in group ID and the fact that they are present in microorganisms in different taxonomic groups, most of them *Alphaproteobacteria* (7/11) but it also includes 1 *Acidobacteria*, 1 *Gammaproteobacteria*, 1 *Betaproteobacteria* and 1 *Bacteroidete* (Additional file 4: Figure S2), strongly suggests some kind of functional conservation of the enzymes encoded by all those genes.

In relation to the possible enzymatic activity of the proteins of groups IB, IC or ID, no oxidase activity has been reported for any of them. However, it has been shown that the proteins from *Caulobacter crescentus* (group IB) and *Chromobacterium violaceum* (group IC) are involved in hydrogen peroxide generation during biofilm development by those microorganisms [11]. Systematic analysis did not allow the detection of lysine oxidase activity in the cultures of those microorganisms as well as in *Marinomonas* sp. MED121 (IB) and *Saccharophagus degradands* 2*–*40 (ID) (Campillo-Brocal et al., unpublished observations). These data suggest that these enzymes oxidize substrates different to L-lysine.

In terms of taxonomic distribution, *Proteobacteria* in general and *Gammaproteobacteria* in particular are highly represented in group I. *Gammaproteobacteria* constitute the 71.4% (15 out of 21) of the microorganisms in the subgroup IA (Figure 4) and 68.4% (13/19) in group IB (Additional file 3: Figure S1). In contrast, they are the 28.4% (41/144) of the total number of microorganisms with genes of the *lodA* family. Interestingly, no *Alphaproteobacteria* is included in group IA and only two belong to group IB, but they are abundant in group ID (7/10). Group IC include microorganisms belonging to different taxonomic groups (Additional file 4: Figure S2).

#### Group II

**Figure 5:**
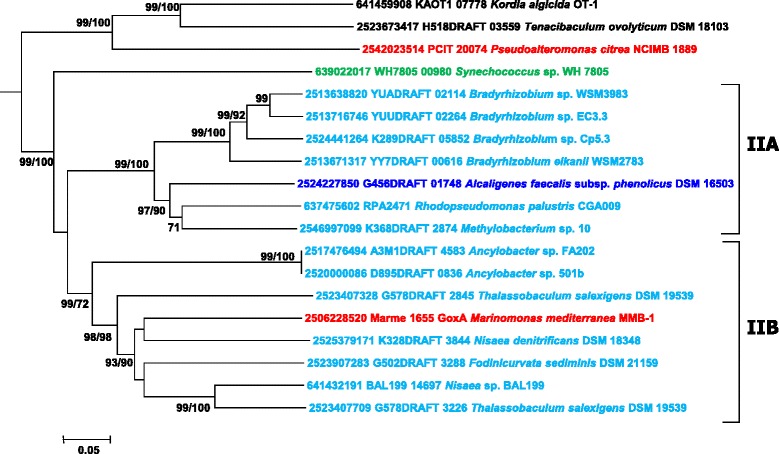
**Phylogenetic relationships of LodA-like proteins in the group II.** The tree was constructed using the Neighbor-Joining method built in the MEGA6 program. The evolutionary distances were computed using the p-distance method and are in the units of the number of amino acid differences per site. Numbers at branches indicate bootstrap values higher than 70% for both, Neighbor-Joining and Maximum Likelihood trees. Color codes for taxonomic groups as in Figure 3.

GoxA, a glycine oxidase synthesized by *Marinomonas mediterranea* [13] clusters in group II of LodA-like proteins (Figure 5). The fact that two *M. mediterranea* proteins, LodA and GoxA, with different enzymatic activities, belong to different groups indicates that the phylogenetic groups described in this study are of interest for exploring the enzymatic activity of the LodA-like proteins. All the proteins in group II, except for the proteins from *Kordia algicida*, *Tenacibaculum ovolyticun* and *Pseudoalteromonas citrea,* could be subdivided in two groups (IIA and IIB) (Figure 5). In terms of protein sequence, an important difference is that the three nonclustered proteins do not show in their N-terminal region a typical twin arginine secretion motif present in all of the others.

It is important to point out that most (13/15) of the proteins of groups IIA and IIB were detected in genomes of *Alphaproteobacteria*. The only two that did not belong to this Class are the betaproteobacterium *Alcaligenes faecalis* subsp. *phenolicus* and the gammaproteobacterium *Marinomonas mediterranea*. In terms of sequence comparison, according to EMBOSS-Needle [31], LodA and GoxA showed 22.8% identity and 34.6% similarity, while GoxA and the LodA-like protein of *Nisaea denitrificans* showed 64.5% identity and 76.4% similarity. These results suggest that *M. mediterranea* could have acquired the *goxA* gene through a process of horizontal gene transfer from an alphaproteobacterium rather than by gene duplication. A process of horizontal gene transfer could have also generated the *lodA*-like gene in *Alcaligenes faecalis* subsp. *phenolicus,* the only betaproteobacterium in this group (Figure 5). In contrast, *Thalassobaculum salexigens* contains two copies of *lodA*-like genes which seem to have been generated by gene duplication, since the two proteins synthesized by this microorganism show 54.8 identity and 66.5 similarity, and cluster together very close in group II (Figure 5).

#### Group III

Group III of LodA-like proteins include a wide range of proteins detected in different microbial groups (Additional file 5: Figure S3)*.* It was possible to detect some subgroups of proteins in group III. Group IIIA contains many proteins synthesized by *Gammaproteobacteria*, while group IIIC only contains *Actinobacteria.* Group III includes all the proteins detected in this study with a fusion to the domain pfam00199 (the catalase domain above mentioned) as well as many other proteins that do not show that fusion. The proteins with domain pfam00199 did not cluster in any subgroup in particular.

As far as we know, no protein in this group has been characterized. Group III includes the product of Marme_2396 which is the third gene of the *lodA* family detected in *Marinomonas mediterranea*. As it has been previously described, the other two genes encode a lysine-ε-oxidase and a glycine oxidase. A double mutant with deletion of *lod* and *gox* operons lost both activities suggesting that the protein in group III shows a different enzymatic activity [13].

In many microorganisms in which more than one copy of *lodA*-like genes have been detected, one of those copies belonged to group III (Table 2). For instance, *Pseudoalteromonas citrea* and *M. mediterranea* were the only two bacteria detected with three copies of genes of the *lodA* family. Interestingly, in both cases the three copies were in the groups I, II and III as defined in this study. This observation suggests that the function of the proteins encoded by those genes could be complementary, perhaps acting on different substrates.

#### Group IV

Group IV is a small group containing just 9 members (Additional file 6: Figure S4). Two subgroups of proteins could be recognized in it. Subgroup IVA contains proteins synthesized by *Alphaproteobacteria* of the order *Rhizobiales*. In subgroup IVB proteins of several bacterial Classes including *Flavobacteria*, *Deltaproteobacteria*, and *Actinobacteria* were included. No protein of this group IV has been characterized so far.

#### Group V

This small group contains the five proteins whose encoding genes were detected in fungi, including the two copies detected in *Gymnopus luxurians* (Additional file 7: Figure S5). These two proteins showed higher similarity between them (60.2% identity and 70.5% similarity) than to any other LodA-like protein detected in this study. In addition, the genes encoding those proteins are close in the fungal genome (Figure 1C). These observations suggest a possible genetic duplication event. As previously discussed, the *lodA*-like genes detected in fungi show an unusual genetic organization since the *lodB*-like gene is oriented in the opposite direction to the *lodA* gene (Figure 1C). In terms of sequence analysis, the LodA-like proteins synthesized by fungi do not show defining features being very similar to the bacterial proteins.

## Conclusions

LodA and GoxA are unconventional amino acid oxidases since they are the first enzymes of this group whose cofactor is CTQ and not FAD [5,6,13]. Genome mining using the sequences of the *M. mediterranea* LodA and GoxA have revealed the presence of 168 genes encoding proteins similar to these two in 144 microbial genomes, representing the 0.91% of all the genomes deposited in IMG database as of January 2014. Many of those genes were annotated as encoding hypothetical proteins, although since the description of LodA, some of them are reported as encoding lysine oxidases. However, as this study reveals, LodA-like proteins can be divided in several clusters and the enzymatic activity may depend on the group considered. For example, *M. mediterranea* GoxA does not show lysine oxidase but glycine oxidase activity [13].

This study provides a platform to analyze the enzymatic activity of novel LodA-like proteins which we consider to be a reservoir of novel enzymatic activities of potential biotechnological interest. Moreover, this study will be very helpful to experimentally address structure-function studies in LodA-like proteins. For example, sequence analysis has revealed several conserved domains and residues in all the proteins analyzed. The functions of most of those domains and residues remain to be analyzed. However, it seems plausible to consider that the conservation of a C and W that align with the C and W in LodA CTQ cofactor indicates that this cofactor is present in all of the proteins detected.

In relation to cofactor biosynthesis, it has been observed that even when a microorganism shows more than one copy of a *lodA*-like gene, each copy is generally followed by a copy of a *lodB*-like gene. This observation suggests that there is a very specific interaction between LodA-like proteins and the flavoproteins encoded in the same operon. In the case of *M. mediterranea* LodA, it has been shown that LodB participates in the generation of the quinone cofactor [9]. Recent studies of our group indicate that in the generation of the cofactor there is a specific interaction between the flavoprotein and the quinoprotein encoded in the same operon [17].

LodA-like proteins seem to have an ancient origin in bacteria since they are present in many different groups. Their evolution in bacterial genomes seems to involve different processes. The data obtained suggest that in some cases, such as the presence of *goxA* in *M. mediterranea,* a process of horizontal gene transfer could have been involved. While in others, such as *T. salexigens,* a genetic duplication event seems to be the most reasonable explanation.

## Methods

### Detection of *lodA*-like and *lodB*-like genes

Most of the analysis performed in this study have been carried out using the tools available at Integrated Microbial Genomes Expert Review (IMG/MER) [32]. Genes encoding proteins similar to LodA with an E-value lower than 1e-10 were identified using BLASTP search using as query the sequences of LodA (accession number AAY33849) and GoxA (accession number ADZ90918). With the sequence of GoxA a few more genes were detected, so this group of genes was selected since it included all the genes detected using LodA sequence as query.

Genes encoding LodB-like proteins were detected in a similar way, using as query LodB sequence (accession number AAY33850). In this case the BLASTP (E-value 1e-10) was limited only to those genomes containing *lodA*-like genes. In most cases, a single hit was obtained against a gene located next to the *lodA*-like gene and this was included in the group of *lodB*-like genes. In fewer cases several hits were obtained, but only the gene close to the *lodA*-like gene (which was the one with the highest score) was selected.

### LodA-like proteins sequence and phylogenetic analysis

All the protein sequences (168) selected in this study were aligned using the program clustal omega [33] available at http://www.ebi.ac.uk/Tools/msa/clustalo/.

Aligned sequences were incorporated into the program MEGA6 [21] to perform the phylogenetic analysis. Two different kind of analysis were performed. First, a tree was constructed using the Neighbor-Joining (NJ) method. In this method the distances between sequences were computed using the p-distance method and are in the units of the number of amino acid differences per site. The reliability of each node in the tree constructed was estimated using bootstrap analysis with 500 replicates.

A second analysis was performed using the Maximum Likelihood (ML) method. To select the most appropriate substitution model in the construction of the tree, the feature “Find Best DNA/Protein Model” incorporated in MEGA was used [34]. The model selected was Le and Gascue (LG) [35]. A discrete Gamma distribution was used to model evolutionary rate differences among sites (5 categories (+G, parameter = 1,3855)). The rate variation model allowed for some sites to be evolutionarily invariable (+I, 4,2775% sites). Pairwise distances were estimated using a Jones-Taylor-Thorton (JTT) model. All positions with less than 95% site coverage were eliminated. That is, fewer than 5% alignment gaps, missing data, and ambiguous bases were allowed at any position. The reliability of each node in the ML tree constructed was estimated using bootstrap analysis with 50 replicates.

The 168 proteins analyzed in this study were associated in groups containing five or more proteins. The criterion followed is that those groups should be supported by bootstrap analysis with higher than 70% reliability in the two phylogenetic analysis performed (NJ and ML).

### Availability of supporting data

The phylogenetic tree with the 168 LodA-like proteins and its associated data matrix are available in TreeBASE database (Accession URL: http://purl.org/phylo/treebase/phylows/study/TB2:S17238). Other supporting data are included as Additional files 1, 2, 3, 4, 5, 6 and 7.

## Additional files

Additional file 1: Table S1. Genes of the *lodA* family detected in IMG database as of January 2014. Additional file 2: Table S2.
*lodB*-like genes encoding putative flavoproteins detected in genomes containing a *lodA*-like gene. Additional genes located between both genes in some genomes are shown in red. Some genes that seem to be a *lodB*-like gene divided in two are shown in blue. Additional file 3: Figure S1. Phylogenetic relationships of LodA-like proteins in the group IB. The tree was constructed using the Neighbor-Joining method built in the MEGA6 program. The evolutionary distances were computed using the p-distance method and are in the units of the number of amino acid differences per site. Numbers at branches indicate bootstrap values > 70% for both Neighbor-Joining and Maximum Likelihood trees. *Gammaproteobacteria* are indicated in red, *Alphaproteobacteria* in light blue, *Betaproteobacteria* in dark blue and photosynthetic microorganisms in green. Additional file 4: Figure S2
**Additional file 4.** Phylogenetic relationships of LodA-like proteins in the group IC and ID. The tree was constructed using the Neighbor-Joining method built in the MEGA6 program. The evolutionary distances were computed using the p-distance method and are in the units of the number of amino acid differences per site. Numbers at branches indicate bootstrap values > 70% for both Neighbor-Joining and Maximum Likelihood trees. *Gammaproteobacteria* are indicated in red, *Alphaproteobacteria* in light blue and *Betaproteobacteria* in dark blue. Asterisks indicate those operons containing a small gene between the *lodA* and *lodB*-like genes. Additional file 5: Figure S3. Phylogenetic relationships of LodA-like proteins in the group III. The tree was constructed using the Neighbor-Joining method built in the MEGA6 program. The evolutionary distances were computed using the p-distance method and are in the units of the number of amino acid differences per site. Numbers at branches indicate bootstrap values > 70 % for both Neighbor-Joining and Maximum Likelihood trees. *Gammaproteobacteria* are indicated in red, *Alphaproteobacteria* in light blue, *Betaproteobacteria* in dark blue and photosynthetic microorganisms in green. Asterisks indicate that the protein shows the conserved domain pfam00199. Additional file 6: Figure S4. Phylogenetic relationships of LodA-like proteins in the group IV. The tree was constructed using the Neighbor-Joining method built in the MEGA6 program. The evolutionary distances were computed using the p-distance method and are in the units of the number of amino acid differences per site. Numbers at branches indicate bootstrap values > 70 % for both Neighbor-Joining and Maximum Likelihood trees. *Alphaproteobacteria* are indicated in light blue. Additional file 7: Figure S5. Phylogenetic relationships of LodA-like proteins detected in fungi (Group V). The tree was constructed using the Neighbor-Joining method built in the MEGA6 program. The evolutionary distances were computed using the p-distance method and are in the units of the number of amino acid differences per site. Numbers at branches indicate bootstrap values > 70% for both Neighbor-Joining and Maximum Likelihood trees.

## Abbreviations

LAO: L-amino acid oxidase
CTQ: Cysteine tryptophylquinone
QHNDH: Quinohemoprotein amine dehydrogenase
BLAST: Basic Local Alignment Search Tool
ML: Maximum Likelihood
NJ: Neighbor-Joining
