# Supplementary figures and images for: Distribution in microbial genomes of genes similar to lodA and goxA which encode a novel family of quinoproteins with amino acid oxidase activity
Source: BMC Genomics. 2015 Mar 24;16(1):231. doi: 10.1186/s12864-015-1455-y (PMC4417212; doi:10.1186/s12864-015-1455-y)

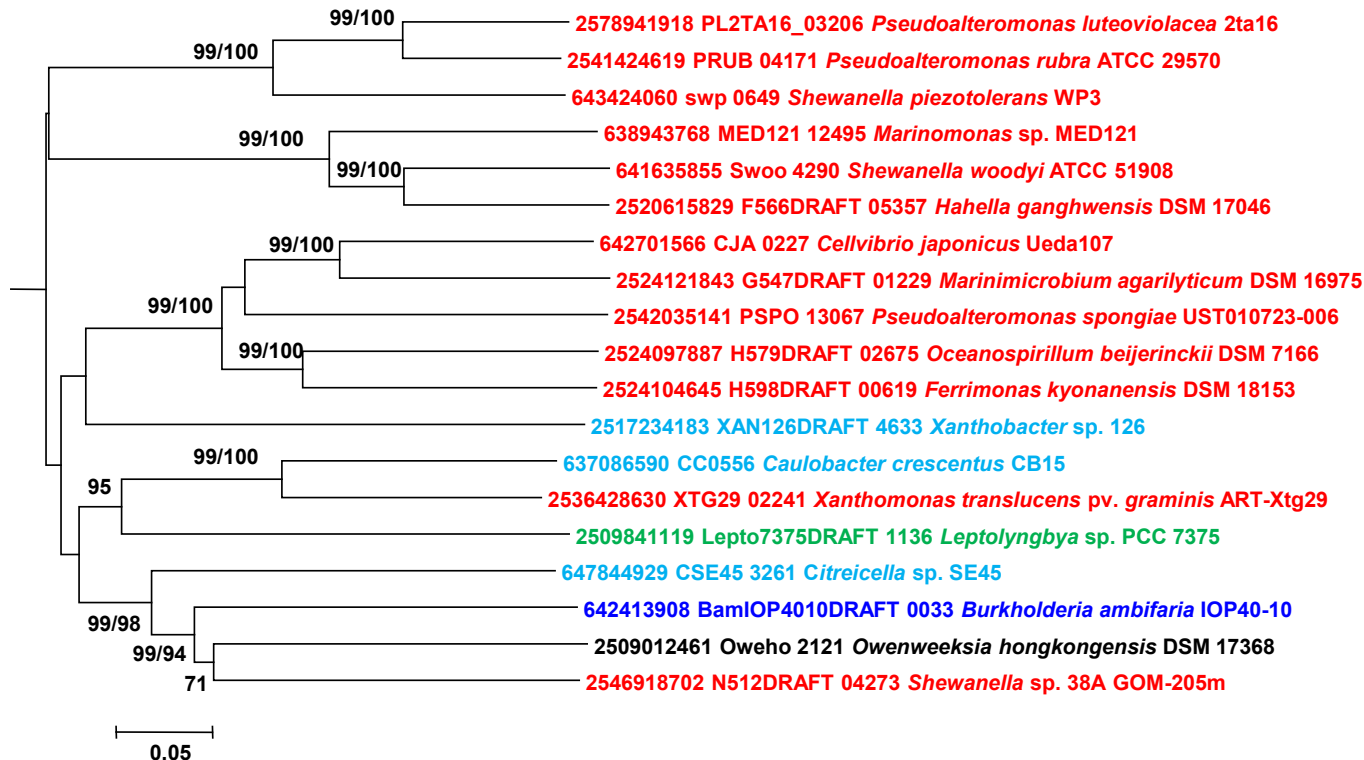

Supplement: Additional file 3: Figure S1. — Phylogenetic relationships of LodA-like proteins in the group IB. The tree was constructed using the Neighbor-Joining method built in the MEGA6 program. The evolutionary distances were computed using the p-distance method and are in the units of the number of amino acid differences per site. Numbers at branches indicate bootstrap values > 70% for both Neighbor-Joining and Maximum Likelihood trees. Gammaproteobacteria are indicated in red, Alphaproteobacteria in light blue, Betaproteobacteria in dark blue and photosynthetic microorganisms in green. [file 12864_2015_1455_MOESM3_ESM.pdf]

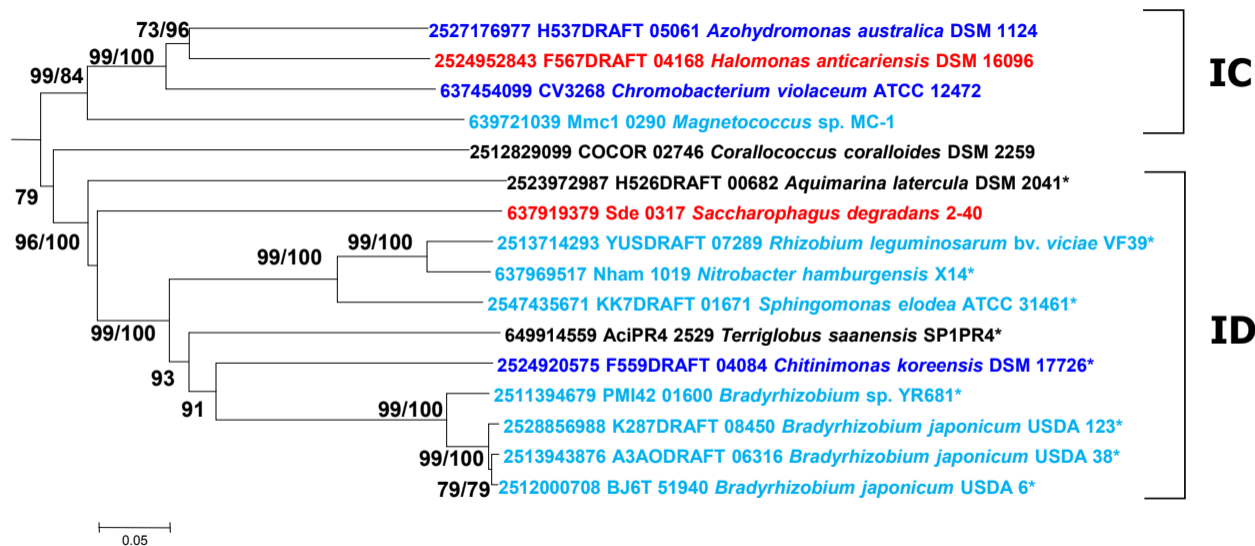

Supplement: Additional file 4: Figure S2 — Additional file 4. Phylogenetic relationships of LodA-like proteins in the group IC and ID. The tree was constructed using the Neighbor-Joining method built in the MEGA6 program. The evolutionary distances were computed using the p-distance method and are in the units of the number of amino acid differences per site. Numbers at branches indicate bootstrap values > 70% for both Neighbor-Joining and Maximum Likelihood trees. Gammaproteobacteria are indicated in red, Alphaproteobacteria in light blue and Betaproteobacteria in dark blue. Asterisks indicate those operons containing a small gene between the lodA and lodB-like genes. [file 12864_2015_1455_MOESM4_ESM.pdf]

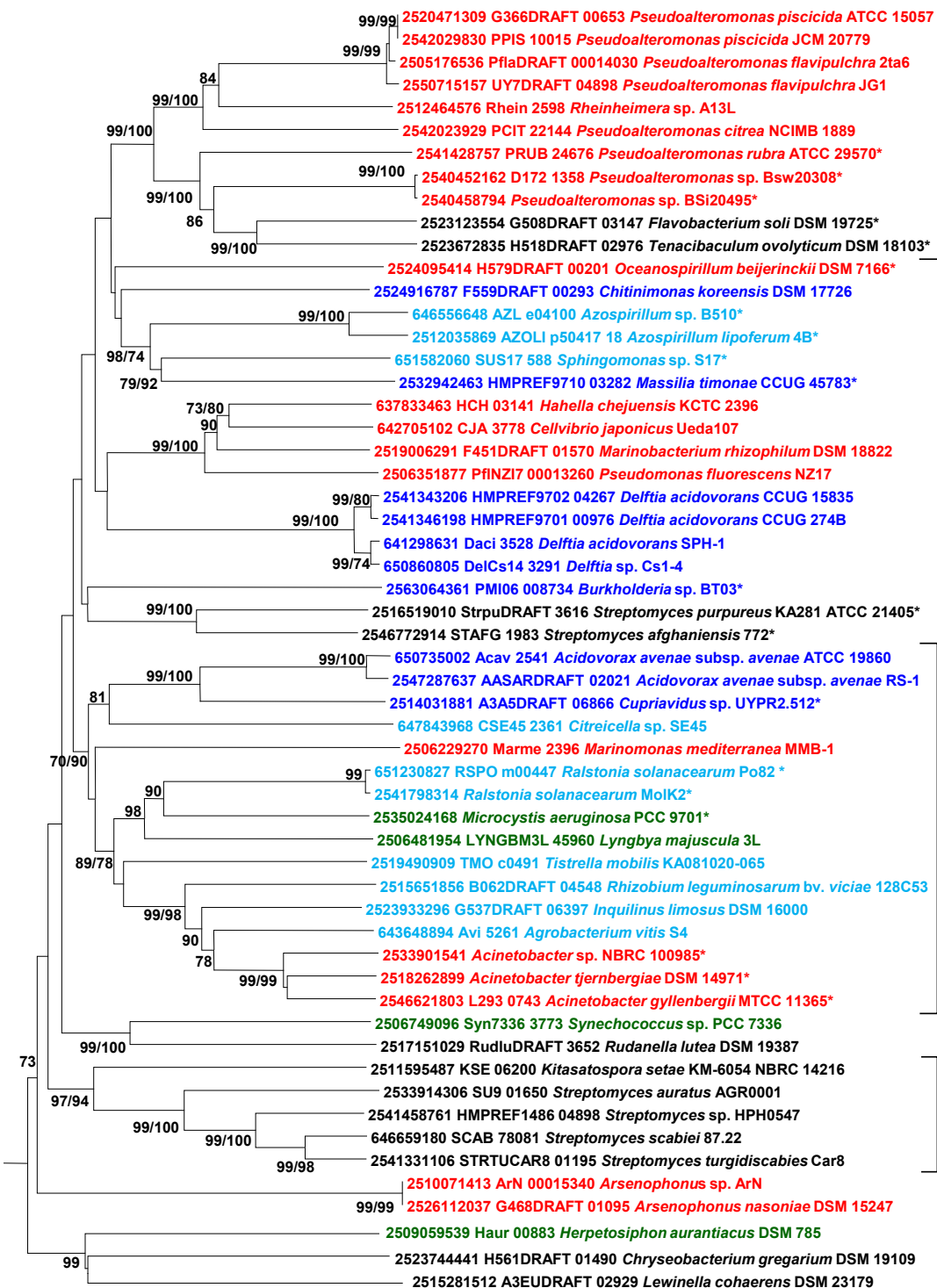

IIIA

IIIB

IIIC

0.05

Supplement: Additional file 5: Figure S3. — Phylogenetic relationships of LodA-like proteins in the group III. The tree was constructed using the Neighbor-Joining method built in the MEGA6 program. The evolutionary distances were computed using the p-distance method and are in the units of the number of amino acid differences per site. Numbers at branches indicate bootstrap values > 70 % for both Neighbor-Joining and Maximum Likelihood trees. Gammaproteobacteria are indicated in red, Alphaproteobacteria in light blue, Betaproteobacteria in dark blue and photosynthetic microorganisms in green. Asterisks indicate that the protein shows the conserved domain pfam00199. [file 12864_2015_1455_MOESM5_ESM.pdf]

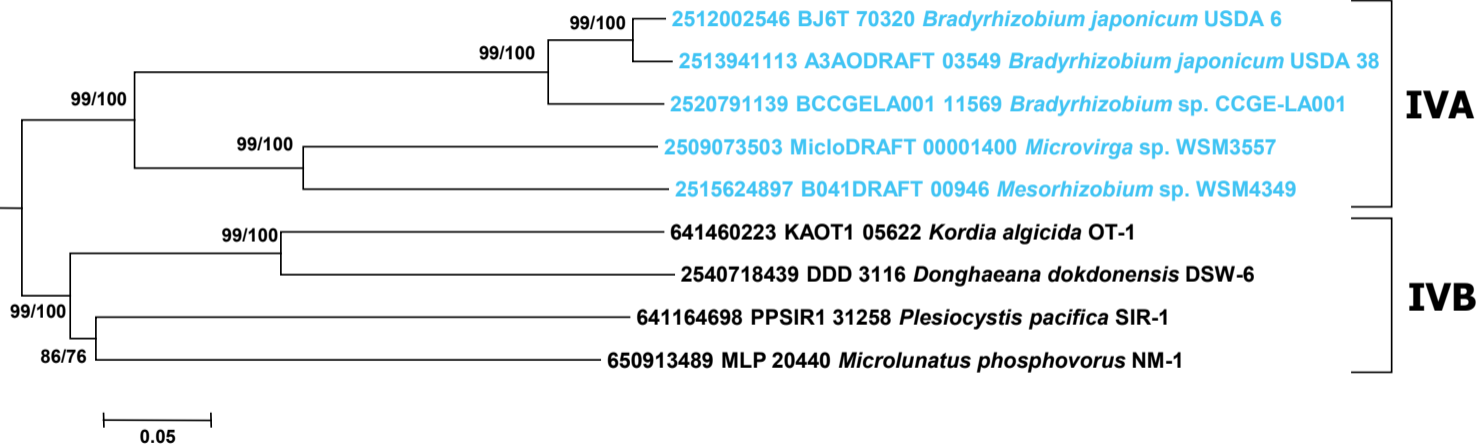

Supplement: Additional file 6: Figure S4. — Phylogenetic relationships of LodA-like proteins in the group IV. The tree was constructed using the Neighbor-Joining method built in the MEGA6 program. The evolutionary distances were computed using the p-distance method and are in the units of the number of amino acid differences per site. Numbers at branches indicate bootstrap values > 70 % for both Neighbor-Joining and Maximum Likelihood trees. Alphaproteobacteria are indicated in light blue. [file 12864_2015_1455_MOESM6_ESM.pdf]

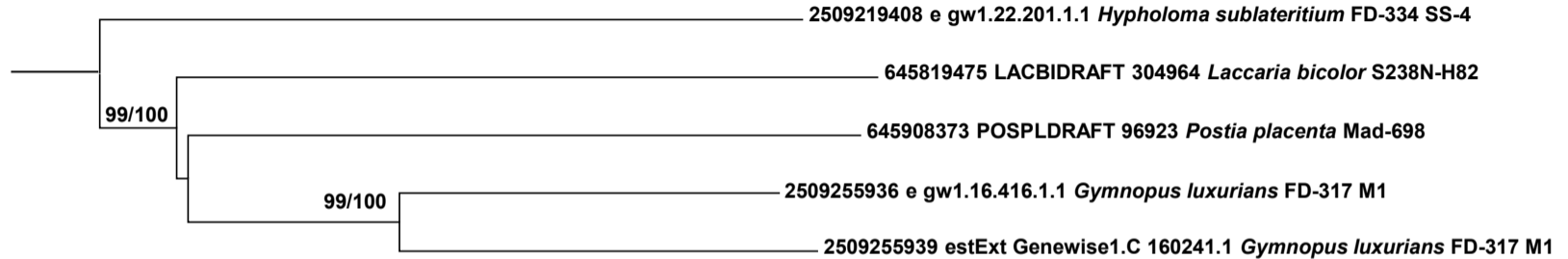

0.05

Supplement: Additional file 7: Figure S5. — Phylogenetic relationships of LodA-like proteins detected in fungi (Group V). The tree was constructed using the Neighbor-Joining method built in the MEGA6 program. The evolutionary distances were computed using the p-distance method and are in the units of the number of amino acid differences per site. Numbers at branches indicate bootstrap values > 70% for both Neighbor-Joining and Maximum Likelihood trees. [file 12864_2015_1455_MOESM7_ESM.pdf]
